# Single Nucleotide Polymorphisms at +191 and +292 of Galectin-3 Gene (*LGALS3*) Related to Lower GAL-3 Serum Levels Are Associated with Frequent Respiratory Tract Infection and Vaso-Occlusive Crisis in Children with Sickle Cell Anemia

**DOI:** 10.1371/journal.pone.0162297

**Published:** 2016-09-07

**Authors:** Taciana Furtado de Mendonça Belmont, Kleyton Palmeira do Ó, Andreia Soares da Silva, Kamila de Melo Vilar, Fernanda Silva Medeiros, Luydson Richardson Silva Vasconcelos, Ana Claudia Mendonça dos Anjos, Betânia Lucena Domingues Hatzlhofer, Maíra Galdino da Rocha Pitta, Marcos André Cavalcanti Bezerra, Aderson da Silva Araújo, Moacyr Jesus Barreto de Melo Rego, Patrícia Moura, Maria do Socorro Mendonça Cavalcanti

**Affiliations:** 1 Programa de Doutorado da Rede Nordeste de Biotecnologia, Recife, Brasil; 2 Instituto de Ciências Biológicas e Faculdade de Ciências Médicas, Universidade de Pernambuco, Recife, Brasil; 3 Laboratório de Imunomodulação e Novas Abordagens Terapêutica (LINAT), Universidade Federal de Pernambuco, Recife, Brasil; 4 Centro de Pesquisas Aggeu Magalhães, CPqAM-FIOCRUZ-PE, Recife, State of Pernambuco, Brazil; 5 Fundação Hematologia e Hemoterapia de Pernambuco (HEMOPE), Recife, Brasil; 6 Departamento de Ciências Biológicas, Universidade Federal de Pernambuco, Recife, Brasil; Universidade de Sao Paulo, BRAZIL

## Abstract

**Introduction:**

Patients with sickle cell anemia (SCA) may present chronic hemolytic anemia, vaso-occlusion and respiratory tract infection (RTI) episodes. Galectin-3 (GAL-3) is a multifunctional protein involved in inflammation, apoptosis, adhesion and resistance to reactive oxygen species. Studies point to a dual role for GAL-3 as both a circulation damage-associated molecular pattern and a cell membrane associated pattern recognition receptor.

**Objective:**

To investigate associations between the SNPs of GAL-3 gene (*LGALS3*) and serum levels with RTI and vaso-occlusive crisis (VOC) in children with SCA.

**Materials and Methods:**

SNPs +191 and +292 in *LGALS3* were studied using the TaqMan real-time PCR system; GAL-3 serum levels were measured by ELISA. The study included 79 children with SCA ranging from 2 to 12 years old.

**Results:**

GAL-3 serum levels were associated with *LGALS3* +191 and +292 genotypes (*p* <0.0001; *p* = 0.0169, respectively). *LGALS3* +191, AA genotype was associated with low and CC with higher levels of GAL-3. For *LGALS3* +292, the CC genotype was associated with lower GAL-3 and AA with higher levels. Patients with Frequency of RTI (FRTI) ≥1 presented higher frequency of +191AA (*p* = 0.0263) and +292AC/CC genotypes (*p* = 0.0320). SNP +292 was associated with Frequency of VOC (FVOC) (*p* = 0.0347), whereas no association was shown with SNP +191 and FVOC. However, CA/AC and AA/CC genotypes with lower GAL-3 levels showed a higher frequency in patients with FRTI ≥1 (*p* = 0.0170; *p* = 0.0138, respectively). Also, patients with FVOC ≥1 presented association with CA/AC (*p* = 0.0228). *LGALS3* +191 and +292 combined genotypes related to low (*p* = 0.0263) and intermediate expression (*p* = 0.0245) were associated with FRTI ≥1. Lower GAL-3 serum levels were associated with FRTI ≥1 (*p* = 0.0426) and FVOC ≥1 (*p* = 0.0012).

**Conclusion:**

Variation of GAL-3 serum levels related to SNPs at +191 and +292 may constitute a susceptibility factor for RTI and VOC frequency.

## Introduction

Sickle-cell anemia (SCA) is a monogenic hemolytic anemia with high phenotypically variable outcome and multisystem pathology [1,2]. Complications are related to polymerization of the abnormal hemoglobin S (HbS), leading to erythrocyte sickling, exposure of membrane proteins, cell adhesion receptors, hemolytic anemia and recurrent ischemia-reperfusion events [3]. The vaso-occlusion corresponds to the most important stimulus for the inflammatory process, due to endothelial dysfunction, increased vascular inflammation, coagulation activation and oxidative stress caused by reinstatement of blood flow [4].

SCA is characterized by painful vaso-occlusive episodes and susceptibility to infections of bacterial, fungal or viral origin [5]. In SCA, inflammation may occur in acute or chronic forms, involving a series of cellular interactions mediated by inflammatory cytokines [6].

Galectin-3 (GAL-3) is one of the most studied members of the galectin family with approximately 30 kDa, characterized by specific binding to β-galactosides [7]. Besides its carbohydrate-recognition domain (CRD), GAL-3 also contains a proline and glycine-rich N-terminal domain, which is able to form oligomers [7–9]. GAL-3 is expressed in a variety of cells and tissues. It can be found both in the nucleus and cytoplasm, at the cell surface or secreted in the extracellular space [7].

GAL-3 is encoded by the *LGALS3* gene, localized on chromosome 14, locus q21–q22. Human *LGALS3* gene is composed of 6 exons and 5 introns [10,11]. The single nucleotide polymorphisms (SNPs) rs4644, *LGALS*3 +191 leads to histidine → proline change at residue 64, whereas rs4652 +292 leads to threonine → proline change at residue 98 of GAL-3 [12]. The *LGALS3* +292 C allele carries proline and was associated with lower serum GAL-3 levels in rheumatoid arthritis (RA) [12], since the proline at GAL-3 residue 98 is located in a critical protein transport determination region [13,14]. Studies on hamsters GAL-3 showed that the short segment of N-terminal sequence residues 89–96 (YPSA***P***GAY) is critical for the secretion of this lectin. In human GAL-3, the residues 94–101 of N-terminal sequence (Y***P***SA***P***GAY) is highly homologous to the hamsters GAL-3 and may also be involved in human GAL-3 secretion [15]. Therefore, SNPs in this region could be involved with the production of different serum levels of GAL-3.

GAL-3 is involved in a variety of biological processes including cellular adhesion, activation, chemotaxis, growth and differentiation, resistance to oxygen and nitrogen radicals damage and apoptosis [16–22]. Moreover, GAL-3 is part of the innate immune response [9,23,24].

Farnworth et al. (2008) [25] suggested that the GAL-3 increased activity could enhance the inflammatory response, since it promotes neutrophil longevity or its bacteriostatic activity, improving clinical outcomes after severe pneumococcal infection.

Recent studies point to a dual role for GAL-3 as circulating damage-associated molecular pattern and cell membrane-associated pattern recognition receptor [26]. Thus, our aim was to investigate the association of SNPs at *LGALS3* and serum GAL-3 levels with Frequency of Respiratory Tract Infection (FRTI) and Frequency of Vaso-occlusive Crisis (VOC) in patients with SCA.

## Material and Methods

### Patients

Seventy-nine children with SCA, aged 2 to 12 years with a median of 5 years (52% males) were studied. The patients enrolled in this study were attended in an ambulatory for hemoglobin disorders and neonatal screening, from 2002 until 2014, diagnosed at the Blood Center of Pernambuco (Hemope Foudation), using electrophoresis of Hemoglobin and High Pressure Liquid Chromatography (HPLC) (BioRad, Hercules, CA, USA). All children were vaccinated against pneumococcus (7 Pneumo-Wyeth Pharmaceuticals Inc. USA) and meningococcus (Meningitec-Wyeth Pharmaceuticals, UK) and used penicillin (4000 U dose/kg/day) prophylactically according to the national protocol.

Epidemiological, clinical and laboratory data were obtained from standardized medical records provided by the Hemope Foundation. Clinical data were considered before initiating treatment with hydroxyurea (HU). For assessment of the GAL-3 serum concentration, children who received a transfusion in the last 3 months were excluded.

The clinical events evaluated were RTI and VOC. The dactylitis and pain crisis (episodes of pain) associated with SCA were designated as VOC. Tonsillitis, bronchitis, pneumonia and bronchopneumonia were designated as RTI. The patients were classified in two groups according to the FVOC and FRTI [27], both determined by the total events observed in the clinical records divided by the age of the child at the end of the study. Patients with frequency ≥1 had one or more events per year (group with severe disease), and those with frequency <1 had less than one event per year (group with mild disease). This project was approved by the Research Ethics Committee of the HEMOPE Foundation, Recife, Pernambuco (registration Nr. 005/2013) and written informed consent was obtained from parents or relatives.

### Samples

Peripheral blood samples were collected into tubes containing 5% ethylenediaminetetraacetic acid (EDTA) as anticoagulant for DNA extraction and tubes without anticoagulant for measurement of GAL-3 serum levels. The biological samples were collected from October 2013 to October 2014. Hematological analysis was performed using an electronic cell counter (STKS, Coulter Corporation, FL, USA), and biochemical analysis was performed using a Roche Cobas Mira Plus Chemistry Analyzer (Roche Diagnostics Corporation, Indianapolis, IN, USA).

### Detection of galectin-3 level by enzyme immunoassay

GAL-3 concentrations were measured in 79 children with SCA using a commercial enzyme-linked immunosorbent assay (ELISA) kit, Human *LGALS3*/Galectin-3 (Sigma Aldrich, USA), according to the manufacturer's instructions. An Epoch microplate reader (Biotek Instruments Inc.) was utilized as apparatus and the Gen5 ELISA program (Biotek^®^) was used to calculate the GAL-3 serum concentrations.

### DNA extraction and *LGALS3* genotyping

The extraction of genomic DNA was performed from peripheral blood using a modified phenol-chloroform technique [28]. Genotyping for SNPs of *LGALS3* gene was performed by real-time PCR using a Rotor Gene 6000 system (Corbett Research Mortlake, Sydney, Australia). The determination of SNPs in regions +191 (rs4644) and +292 (rs4652) of *LGALS3* were performed using the Taqman^®^ Genotyping methodology assays C___7593635_1_ and C___7593636_30, respectively.

### Statistical analysis

To compare categorical variables between the groups, we used a chi-square test (χ^2^) with Yates correction or Fisher's exact test when necessary. The association between the variables was estimated by the odds ratio (OR) with a 95% confidence interval (CI), considering significant *p* <0.05. The D'Agostino-Pearson test was used to assess the normal distribution of quantitative variables. For comparison of these variables between two groups, a Student’s t test or nonparametric Mann-Whitney test were applied. For three or more groups, the Kruskal-Wallis test or ANOVA were applied when appropriate. GraphPad PRISM software Version 5.0) for Windows (GraphPad Software, San Diego, California, USA) was used for these analyses. Haploview software (version 4.2) was used to test the Hardy-Weinberg equilibrium.

## Results

### *LGALS3* SNPs

Regarding patient’s characteristic and the frequency distribution of *LGALS3* +191 and +292 SNPs genotypes, no difference was observed (*p* >0.05) (Table 1). The frequency of the ABO blood group didn’t differ in relation to the genotypes of *LGALS3* +191 and +292 SNPs. Besides, the distribution of the ABO group showed no difference concerning the FRTI or FVOC (Table A and B in S1 File).

**Table 1:** Clinical data of children with sickle cell anemia attended in Hemope Foundation—Recife/Brazil.

	*LGALS3* +191				*LGALS3* +292			
	CC	AC	AA		AA	AC	CC	
	(N = 40)	(N = 32)	(N = 07)	*p*-value	(N = 20)	(N = 36)	(N = 23)	*p*-value
Gender—n (%)								
Male	18 (67.9)	17 (43.1)	06 (43.1)	-	11 (60.5)	16 (41.7)	14 (41.7)	-
Female	22 (32.1)	15 (56.9)	11 (56.9)	0.4834	09 (39.5)	20 (58.3)	09 (58.3)	0.4449
Age—Years								
Median (Min–Max)	05 (02–10)	06 (03–11)	05 (03–11)	0.6910	05 (02–11)	07 (02–12)	05 (03–11)	0.0840
Laboratory Data								
Median (Min–Max)								
Leukocytes (x10^3^/mm^3^)	15.3 (5.8–21.7)	14.3 (1.8–48.2)	12.7 (6.3–22.6)	0.3699	15.6 (7.2–21.4)	14.3 (5.8–21.7)	14.3 (5.8–21.7)	0.3385
Platelets (x10^3^/mm^3^)	441 (169–785)	431 (206–670)	343 (190–618)	0.3700	441 (169–764)	431 (231–785)	367 (190–670)	0.4673
Hb (g/dL)	7.6 (5.4–12.0)	8.2 (6.5–10.6)	7.6 (6.8–8.8)	0.4811	8.0 (5.4–10.0)	7.5 (6.1–12.0)	8.0 (6.5–10.6)	0.9306
LDH (U/L)	897 (211–2999)	746 (404–1499)	1285 (727–1415)	0.3127	754 (211–2999)	907 (401–2058)	786 (409–1491)	0.8673
TB (μmol/L)	1.7 (0.4–5.6)	1.5 (0.4–4.5)	1.7 (1.4–2.0)	0.9795	1.6 (0.9–3.2)	1.8 (0.4–4.5)	1.5 (0.6–5.6)	0.5611
UB (μmol/L)	1.3 (0.3–4.9)	1.1 (0.2–4.3)	1.3 (1.0–1.6)	0.9762	1.2 (0.7–3.2)	1.4 (0.2–4.3)	1.1 (0.4–4.9)	0;5915
Ret (%)	9.4 (1.1–18.3)	9.4 (4.4–22.4)	10.4 (6.4–15.9)	0.5450	12.5 (4.2–18.3)	8.1 (1.1–22.2)	10.1 (5.1–17.6)	0.0557

*LGALS3* +191: C = reference alelle; A = variant allele; *LGALS3* +292: A = reference allele; C = variant allele; SCA = sickle cell anemia; Age, Leukocytes, Hemoglobin (Hb), Platelets, Percent of Reticulocytes (Ret) Lactate Dehydrogenase (LDH), Total Bilirubin (TB), Unconjugated Bilirubin (UB). Mann-Whitney tests.

Genotypes and allele frequencies of *LGALS3* +191 and +292 SNPs in children with SCA are shown in Table 2. Children with SCA were in Hardy–Weinberg equilibrium. The FTRI and FVOC were evaluated in relation to frequencies of *LGALS3* SNPs (Table 2). A positive association was found between children with FRTI >1 and +191AA genotype (lower serum GAL-3 level), when compared to +191CC genotype (higher serum GAL-3 levels) (*p* = 0.026, OR = 7.50, CI = 1.25 to 44.90). There was also an association with +191 A allele and FRTI ≥1 (*p* = 0.018, OR = 2.39, CI = 1.81 to 4.85). However, +191 SNP showed no association with FVOC. For +292 SNP, an association between FRTI ≥1 and +292 AC (*p* = 0.023 OR = 4.22, CI = 1.32 to 13.8) or +292 AC+CC genotypes (intermediate/low serum GAL-3 levels) (*p* = 0.032, OR = 4.17, CI = 1.10 to 15.78) was observed, when compared to +292 AA genotype (higher serum GAL-3 levels). Thus, genotypes of GAL-3 related to low or intermediate serum levels seem to be associated to RIT and VOC in children with SCA.

**Table 2:** *LGALS3* +191 and +292 with vaso-occlusive crisis and respiratory tract infection in children with sickle cell anemia attended in Hemope Foundation—Recife/Brazil.

*LGALS3* +191	SCA	FRTI ≥1	FRTI <1		FVOC ≥1	FVOC <1	
Genotype	(N = 79)	(N = 28)	(N = 51)	*p-*value	(N = 43)	(N = 36)	*p-*value
CC	40 (0.51)	10 (0.36)	30 (0.59)	-	21 (0.49)	19 (0.53)	-
CA	32 (0.40)	13 (0.46)	19 (0.37)	0.206	17 (0.39)	15 (0.42)	1.000
AA	07 (0.09)	05 (0.18)	02 (0.04)	**0.026**	05 (0.12)	02 (0.05)	0.436
CA+AA	39 (0.49)	18 (0.64)	21 (0.41)	0.062	22 (0.51)	17 (0.47)	0.882
**Alelle**							
C	112 (0.71)	33 (0.59)	79 (0.77)	-	59 (0.69)	53 (0.74)	-
A	46 (0.29)	23 (0.41)	23 (0.23)	**0.018**	27 (0.31)	19 (0.26)	0.598
***LGALS3* +292**							
**Genotype**							
AA	20 (0.25)	03 (0.11)	17 (0.33)	-	07 (0.16)	13 (0.36)	-
AC	36 (0.46)	16 (0.57)	20 (0.39)	**0.039**	25 (0.58)	11 (0.31)	**0.023**
CC	23 (0.29)	09 (0.32)	14 (0.28)	0.099	11 (0.26)	12 (0.33)	0.537
AC+CC	59 (0.75)	25 (0.89)	34 (0.67)	**0.032**	36 (0.84)	23 (0.64)	0.068
**Alelle**							
A	76 (0.48)	22 (0.39)	54 (0.53)	-	39 (0.45)	37 (0.51)	-
C	82 (0.52)	34 (0.61)	48 (0.47)	0.134	47 (0.55)	35 (0.49)	0.523

*LGALS3* +191: C = reference allele; A = variant allele; *LGALS3* +292: A = reference allele; C = variant allele; SCA = sickle cell anemia; FRTI = Frequency of Tract Respiratory Infection; FVOC = Frequency of vaso-occlusive crisis. Chi-squared *test* with the Yates correction (OR CI 95%).

The combination of SNPs +191 and +292 generated six possible *LGALS3* diplotypes. The distribution of the CA/AC and AA/CC diplotypes were significantly higher in the FRTI ≥1 group, when compared to the CC/AA diplotype (*p* = 0.0170, OR = 6.67, CI = 1.42 to 31.24; *p* = 0.0138, OR = 13.33, CI = 1.71 to 103.80, respectively). On the other hand, the FVOC ≥1 group showed an association with the CA/AC diplotype, in comparison to CC/AA (*p* = 0.0489, OR = 4.45, CI = 1.11 to 17.90) (Table 3).

**Table 3:** *LGALS3* +191 and +292 combined genotype distribution with vaso-occlusive crisis and respiratory tract infection in children with sickle cell anemia attended in Hemope Foundation—Recife/Brazil.

*LGALS3*	SCA	FRTI ≥1	FRTI <1		FVOC ≥1	FVOC <1	
+191/+292	(N = 79)	(N = 28)	(N = 51)	*p-*value	(N = 43)	(N = 36)	*p-*value
CC/AA	19 (0.24)	03 (0.11)	16 (0.31)	-	07 (0.16)	12 (0.33)	-
CC/AC	19 (0.24)	06 (0.21)	13 (0.25)	0.447	12 (0.28)	07 (0.19)	0.194
CC/CC	02 (0.03)	01 (0.04)	01 (0.02)	0.352	02 (0.05)	00 (0.00)	0.171
CA/AC	18 (0.23)	10 (0.35)	08 (0.16)	**0.017**	13 (0.30)	05 (0.14)	**0.049**
CA/CC	14 (0.18)	03 (0.11)	11 (0.22)	1.000	04 (0.09)	10 (0.28)	0.719
AA/CC	07 (0.08)	05 (0.18)	02 (0.04)	**0.014**	05 (0.12)	02 (0.06)	0.190

*LGALS3* +191: C = reference allele; A = variant allele; *LGALS3* +292: A = reference allele; C = variant allele; SCA = sickle cell anemia; FRTI = Frequency of Tract Respiratory Infection; FVOC = Frequency of vaso-occlusive crisis. Chi-squared *test* with Yates correction (OR CI 95%).

We then grouped the combined genotypes according to the serum GAL-3 levels, establishing a high (CC/AA), an intermediate (CC/AC; CA/AC; CC/CC) and a low (AA/CC; CA/CC) diplotype. A positive association was found between FRTI ≥1 and intermediate genotypes of GAL-3 levels compared to genotypes of higher levels (*p* = 0.0438, OR = 4.12, CI = 1.03 to 16.49). Also, intermediate GAL-3 levels genotypes where more frequent in the group with FVOC ≥1 compared to genotypes of higher levels (*p* = 0.0250, OR = 3.86, CI = 1.22 to 12.23) (Table 4).

**Table 4:** *LGALS3* +191 and +292 diplotype of serum levels with vaso-occlusive crisis and respiratory tract infection in children with sickle cell anemia attended in Hemope Foundation—Recife/Brazil.

*LGALS3*	SCA	FRTI ≥1	FRTI <1		FVOC ≥1	FVOC <1	
	(N = 79)	(N = 28)	(N = 51)	*p-*value	(N = 43)	(N = 36)	*p-*value
High	19 (0.24)	03 (0.11)	16 (0.31)	-	07 (0.16)	12 (0.34)	-
Intermediate	39 (0.49)	17 (0.61)	22 (0.43)	**0.044**	27 (0.63)	12 (0.33)	**0.025**
Low	21 (0.26)	08 (0.28)	13 (0.26)	0.163	09 (0.21)	12 (0.33)	0.755
Intermediate/Low	60 (0.76)	25 (0.89)	35 (0.69)	0.054	36 (0.84)	24 (0.66)	0.113

Diplotype according to the serum GAL-3 level: high (CC/AA), intermediate (CC/AC; CA/AC; CC/CC), low (AA/CC; CA/CC). SCA = sickle cell anemia; FRTI = Frequency of Tract Respiratory Infection; FVOC = Frequency of vaso-occlusive crisis. Chi-squared *test* with Yates correction (OR CI 95%).

### Serum Galectin-3 levels and *LGALS3* +191 and +292 SNPs

**Fig 1:**
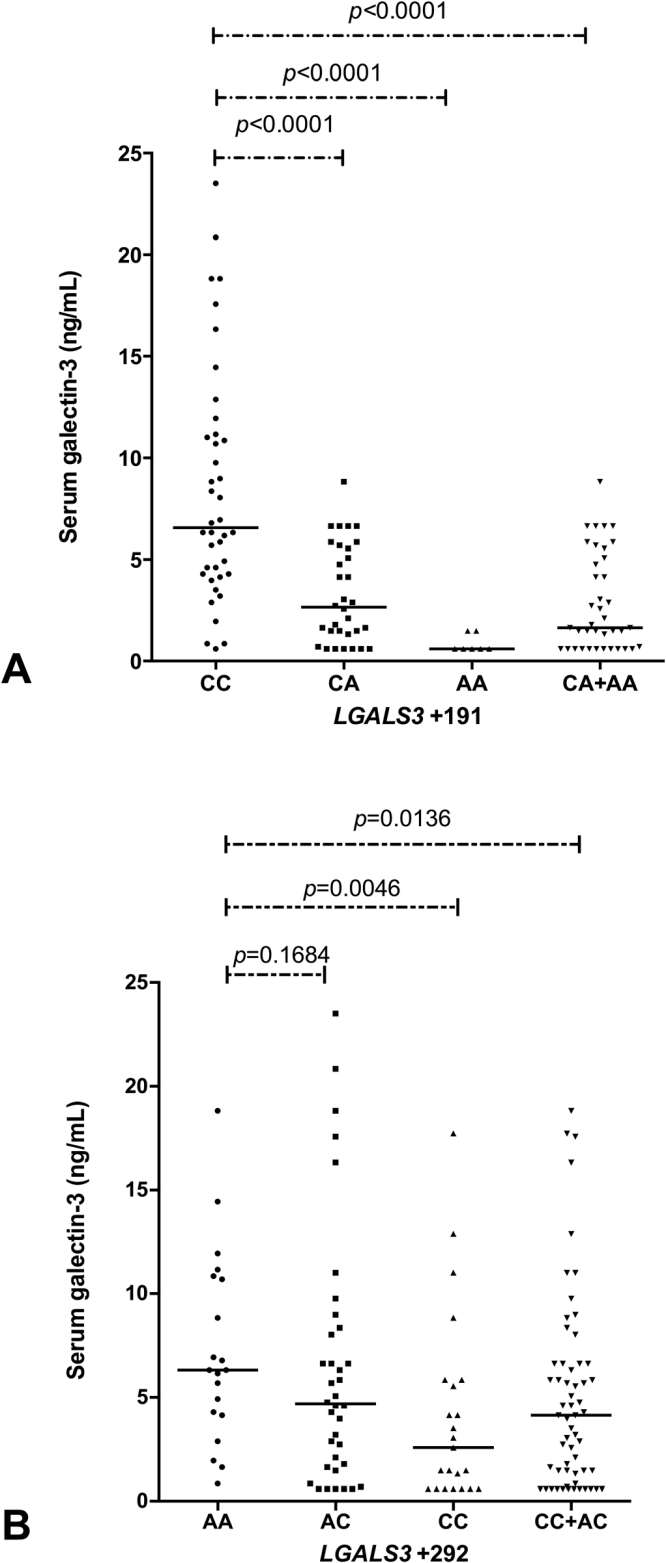
Serum galectin-3 levels associated with *LGALS3* +191 (A) and +292 (B) genotypes in children with SCA. *LGALS3* +191: C = reference allele; A = variant allele; *LGALS3* +292: A = reference allele; C = variant allele; +191 genotypes: CC vs. CA: *p <*0.0001; CC vs. AA: *p <*0.0001; CC vs. AA+CA: *p* <0.0001. +292 genotypes: AA vs. AC: *p* = 0.1684; AA vs. CC: *p* = 0.0046; AA vs. CC+AC: *p* = 0.0136. Mann-Whitney tests.

Serum GAL-3 levels were associated to *LGALS3* +191 and +292 genotypes (*p* <0.0001; *p* = 0.0169, respectively). *LGALS3* +191 CC was associated with high levels (6.56 [0.60 to 23.52] ng/ml, n = 40), followed by CA (2.66 [0.60 to 8.83] ng/ml, n = 32) and finally the AA genotype with lower GAL-3 levels (0.60 [0.60 to 1.48] ng/m, n = 07) (Fig 1A). *LGALS3* +292 AA genotype was associated with high levels (6.33 [0.86 to 18.83] ng/ml, n = 20), followed by AC (4.69 [0.60 to 23.52] ng/m, n = 36) and CC genotype (2.58 [0.60 to 17.73] ng/ml, n = 23) was associated to lower GAL-3 levels (Fig 1B).

**Fig 2:**
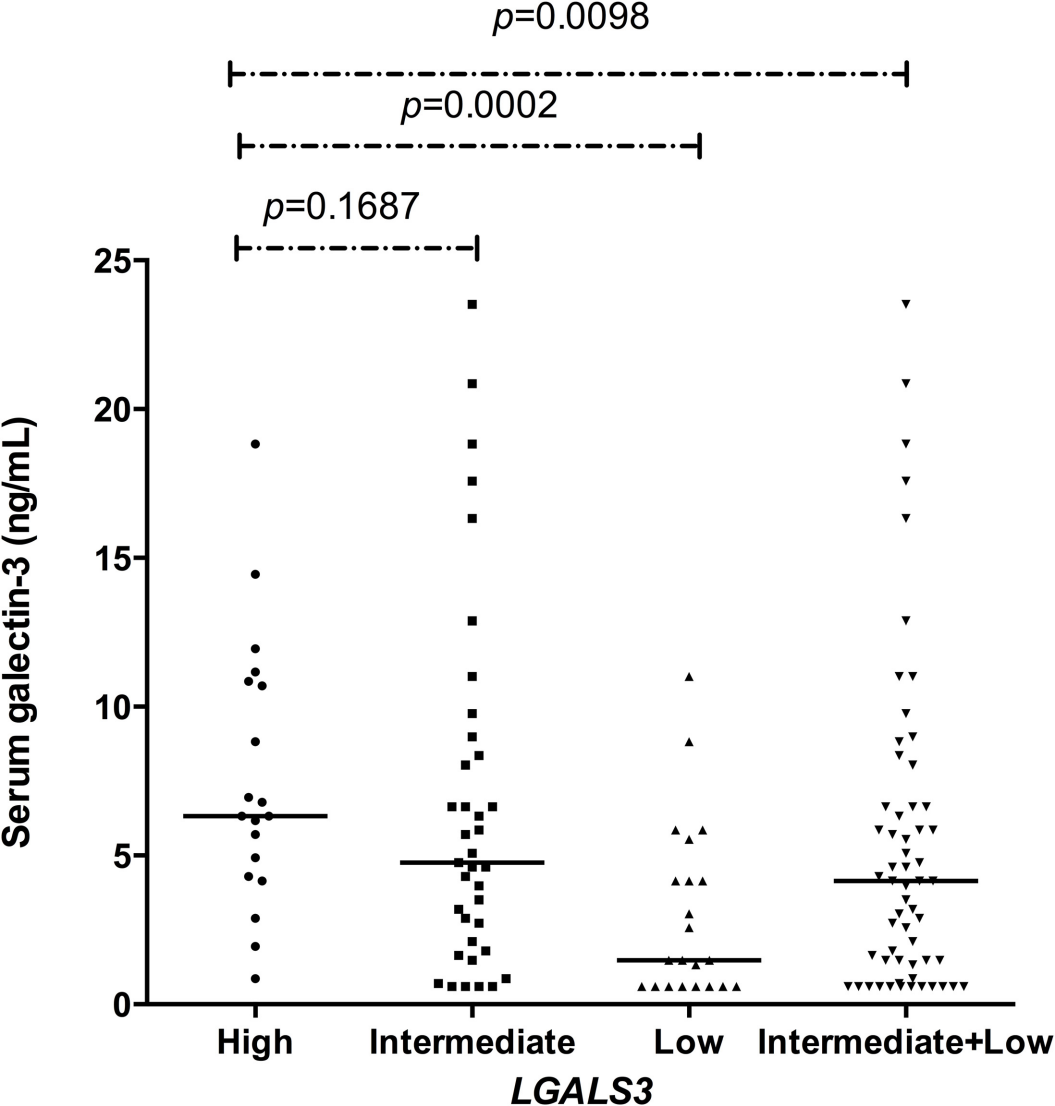
Serum galectin-3 levels are associated with *LGALS3* +191 and +292 combined genotypes in children with SCA. Statistical analysis of *LGALS3* combined genotype related of galectin-3 levels (high, intermediate and low) in which the serum concentrations in ng/ml were compared using Kruskal-Wallis test. High vs. intermediate: *p =* 0.1687; high vs. low: *p =* 0.0002; high vs. intermediate+low: *p* = 0.0098.

There was a significant difference in the serum GAL-3 levels between the combined *LGALS3* genotypes, which were categorized into three groups associated with high (6.33 [0.86 to 18.83] ng/ml, n = 19), intermediate (4.77 [0.60 to 23.52] ng/ml, n = 37) and low (1.48 [0.60 to 11.02] ng/ml, n = 22) serum GAL-3 levels (*p* = 0.00027) (Fig 2).

**Fig 3:**
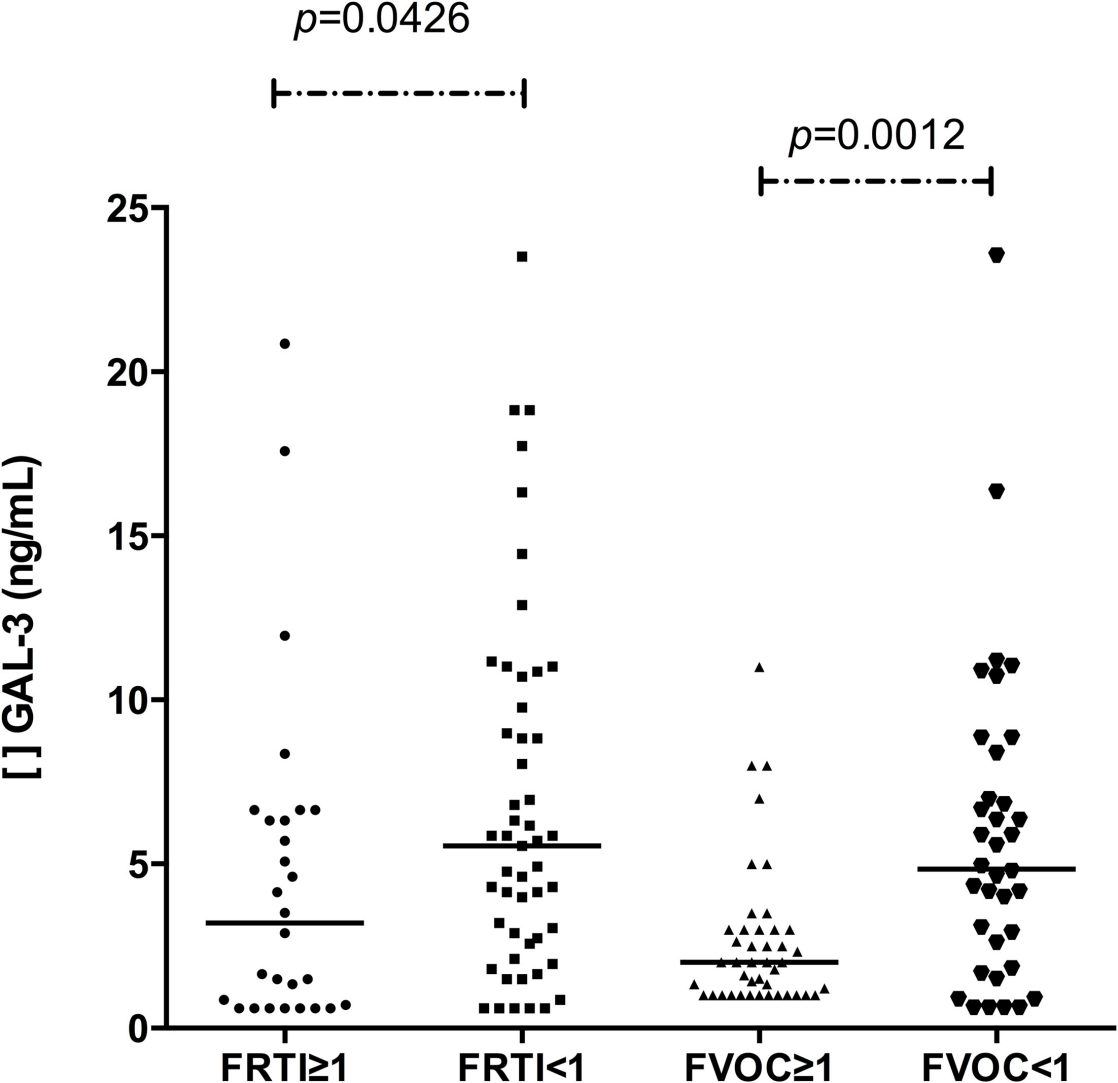
Serum galectin-3 levels associated with frequency of respiratory tract infection (FRTI) and vaso-occlusive crisis (FVOC) in children with SCA. FRTI ≥1 vs. FRTI <1: *p* = 0.0426. FVOC ≥1 vs. FVOC <1: *p* = 0.0012. Mann-Whitney tests.

There was an association between lower serum GAL-3 levels and the FRTI ≥1 group [3.20 (0.60 to 20.86) ng/ml; n = 28], when compared with the FRTI <1 group [5.55 (0.60 to 23.53) ng/ml; n = 51], (*p* = 0.0426). Likewise, there was an association between lower levels of GAL-3 and the FVOC≥1 group [2.0 (1.0 to 11.0) ng/ml; n = 43], when compared to the FVOC <1 group [4.85 (0.60 to 23.52) ng/ml; n = 36], (*p* = 0.0012) (Fig 3).

## Discussion

The contribution of a set of genetic variations such as SNPs can be an explanation for the clinical diversity presented in different populations. In this study, we found an association between the *LGALS3* SNPS related to intermediate serum GAL-3 levels and FRTI and FVOC in children with SCA. Also, low and intermediate serum GAL-3 levels were associated with FRTI and FVOC.

Serum GAL-3 levels in children with SCA varied according to *LGALS3* SNPs (Figs 1 and 2). This is the first study to our knowledge that evaluated the association of serum GAL-3 levels with *LGALS3* SNPs in SCA.

Since the population in Brazil has mixed-race ancestry (European, African and Amerindian contribution [29]), the patients in our study were matched by age, gender and geographical region to minimize stratification bias (Table 1). Epidemiological and clinical parameters were not different when the population was grouped by +191 and +292 *LGALS3* SNPs genotypes (Table 1). This finding may reflect the patients’ basal clinical status at blood sample collection, showing no active crisis. ABO blood group also didn’t differ when analyzed in relation to the genotypes and FRTI or FVOC, that could influence the levels since the GAL-3 binds to the N-acetylgalactosamine, whereas this residue is present in the erythrocytes of the A group.

Regarding all population, +191 SNP presented allelic frequency of 0.71 C and 0.29 A; for the +292 SNP it was 0.52 C and 0.48 A (Table 2). The allelic frequency at +191C, herein reported for patients with SCA, was similar to that observed for subjects with RA (0.82) and controls (0.84) in Taiwan population [12].

In regards to the +191 and +292 combined genotypes it was found a higher frequency of intermediate/low serum levels genotypes with FRTI and FVOC (Tables 3 and 4). Hu *et al*. (2011) [12], analyzing GAL-3 in patients with RA, reported that patients with *LGALS3* +292 CC/CA genotypes had decreased serum GAL-3 levels, compared to AA genotype (*p* = 0.006). Increase of intracellular GAL-3 expression is involved in angiogenesis, cytokines and chemokines fibroblast expression and apoptosis inhibition of inflammatory cells. Those authors suggested that the presence of +292 C allele related to intermediate/low GAL-3 levels would be a factor for susceptibility to RA. They hypothesized that low serum GAL-3 levels may influence the persistence of T cell and macrophage at RA synovium inflammatory site [12].

Additionally, GAL-3 seems to play a critical role in phagocytosis of opsonized erythrocytes [30]. Sano *et al*. (2003) [30] found that GAL-3 is essential for effective phagocytosis for IgG-opsonized erythrocytes and apoptotic cells *in vitro* and *in vivo*.

An interesting study to be conducted could be the investigation of GAL-3 binding preference to ABO blood group in relation to clinical outcome in SCA, since the N-acetylgalactosamine is the principal antigen on erythrocytes of A blood group. Therefore, the sickled cell of A blood group would be removed with more efficiency than others. On the other hand, GAL-3 could agglutinate more the A blood group erythrocytes, compared to B and O groups, worsening the inflammation and favouring the VOC. Our data showed that serum GAL-3 levels were lower in patients of A blood group compared to AB, B or O (*p* = 0.0205). Nevertheless, the present study wasn’t designed to investigate this aspect. Thus, we can’t rule out a sinergic effect of the ABO blood group in the SNPs +191 and +292 of GAL-3 and SCA pathogenesis.

Patients with SCA have a chronic inflammation, characterized by ischemic condition and recurrent reperfusion, leading to the generation of oxidative burst and endothelial activation, as well as upregulation of adhesion molecules [31–33]. Therefore, GAL-3 may be important to SS erythrocyte removal and adhesion inhibition of those sickled erythrocytes in postcapillary venules protecting from VOC processes [30]. Regarding our findings for +292 C, this allele and genotypes AC/CC were more frequent in children with FRTI and FVOC (*p* = 0.032, *p* = 0.023, respectively). Therefore, the same could be reasoned to patients with SCA, since RTI and VOC present an important inflammatory feature, which may involve GAL-3.

Besides its role in regulation of inflammation, GAL-3 may also influence the adaptative immune response. Secreted GAL-3 can cross-link surface glycoproteins and activate pathways involved in several innate immune responses, such as the oxidative burst in neutrophils [34]. GAL-3 (-/-) mice develop more severe pneumonia after infection with *Streptococcus pneumoniae*, showing bacteremia and lung damage compared to wild-type mice [25].

Further, it was found that GAL-3 reduces the severity of pneumococcal pneumonia, among others by increasing the neutrophil function [25]. These authors showed that the GAL-3 directly acts as a neutrophil-activating agent and potentiates the effect of formyl-methionil-leucyl-phenylalanine; the exogenous GAL-3 enhances neutrophil phagocytosis of bacteria and delays neutrophil apoptosis. Moreover, GAL-3 (-/-) macrophages have less efficient phagocytosis of apoptotic neutrophils compared to wild type. Then, GAL-3 is a key molecule in the host defense against pneumococcal infection [25].

Although all patients in this study had received penicillin and conjugated vaccines as routine prophylaxis against *S*. *pneumoniae*, the success of this procedure depend upon cellular effector mechanisms such as phagocytosis. Therefore, our results suggest that children with SCA and low serum GAL-3 levels or carrying *LGALS3* intermediate/low serum genotypes may have disvantages in the defense against pneumococcal infections reflected by the higher FRTI in the children with SCA.

## Conclusion

This study demonstrated an association between *LGALS3* related to intermediate/low serum GAL-3 levels with higher FRTI and FVOC in children with SCA, as well as the serum GAL-3 levels directly. Therefore, the polymorphism of *LGALS3* could be considered as genetic marker for predisposition of RTI and VOC. However, the genetic frequencies of SNPs should be limitated to the studied population due to the small sample size. Nevertheless, the combined analysis of SNPs and serum levels strongly suggests that these SNPs could be of great importance in the variation of serum levels and consequently influencing the pathogenesis of SCA.

Furthermore, studies designed to investigate the influence on the increase of GAL-3 expression and/or its levels are needed and could support the development of new treatments, modulating severity of the SCA. Another important aspect would be to elucidate the interaction of GAL-3 with sickled erythrocytes and its ability to modulate inflammation in patients with SCA.

## Supporting Information

S1 File Table A. Distribution of the ABO group regarding to the FRTI and FVOC. Table B. Genotypes of *LGALS3* +191 and +292 and blood group distribuition.
(DOCX)
