# Supplementary material for: Single Nucleotide Polymorphisms at +191 and +292 of Galectin-3 Gene (LGALS3) Related to Lower GAL-3 Serum Levels Are Associated with Frequent Respiratory Tract Infection and Vaso-Occlusive Crisis in Children with Sickle Cell Anemia
Source: PLoS One. 2016 Sep 7;11(9):e0162297. doi: 10.1371/journal.pone.0162297 (PMC5014331; doi:10.1371/journal.pone.0162297)
Supplement: S1 File — Table A. Distribution of the ABO group regarding to the FRTI and FVOC. Table B. Genotypes of LGALS3 +191 and +292 and blood group distribuition. (DOCX) [file pone.0162297.s001.docx]

**Supporting information**

**S1 Table A- Distribution of the ABO group regarding to the FRTI and FVOC.**

| **ABO blood group** | **FRTI ≥1 (n=25)** | **FRTI <1 (n=40)** | ***p*-value** | **FVOC ≥1 (n=34)** | **FVOC ≤ 1 (n=31)** | ***p*-value** |
| --- | --- | --- | --- | --- | --- | --- |
| **A** | 08 (32) | 12 (30) |  | 12 (35) | 08 (26) |  |
| **B** | 01 (04) | 07 (18) | 0.2651 | 05 (15) | 03 (10) | 0.4947 |
| **O** | 16 (64) | 21 (52) |  | 17 (50) | 20 (64) |  |

**S1 Table B - Genotypes of *LGALS3* +191 and +292 and blood group distribuition.**

| **ABO blood group**  ***LGALS3*** | **A**  **N=20 (%)** | **B**  **N=08 (%)** | **O**  **N=37 (%)** | ***p*-value** |
| --- | --- | --- | --- | --- |
| **+191** |  |  |  |  |
| CC | 10 (50) | 04 (50) | 19 (51) |  |
| CA | 09 (45) | 04 (50) | 13 (35) |  |
| AA | 01 (05) | 00 (00) | 05 (14) |  |
| CA+AA | 10 (50) | 04 (50) | 18 (49) | 0.9942 |
| **+292** |  |  |  |  |
| AA | 05 (25) | 02 (26) | 09 (24) |  |
| AC | 12 (60) | 03 (37) | 17 (46) |  |
| CC | 03 (15) | 03 (37) | 11 (30) |  |
| AC+CC | 15 (75) | 06 (74) | 28 (76) | 0.9980 |
